# The effect of domain and framing on elicited risk aversion

**DOI:** 10.1371/journal.pone.0267696

**Published:** 2022-09-26

**Authors:** María Paz Espinosa, Lara Ezquerra

**Affiliations:** 1 Department of Economics, University of the Basque Country, Bilbao, Spain; 2 Department of Business Economics, University of the Balearic Islands, Palma de Mallorca, Spain; Universidad Pablo de Olavide, SPAIN

## Abstract

Experimental literature has found that risk attitudes are not robust to different elicitation techniques. However, most comparisons across elicitation methods involve different rewards and framings simultaneously. Our experimental design helps to disentangle the effect of these two factors. We consider two different personal rewards (*money domain* and *grade domain*) and two different scenarios while keeping the reward constant (*lottery framing* and *exam framing*). We find no differences in elicited risk aversion between the two domains. However, framing matters: elicited risk aversion is lower in the exam framing.

## 1. Introduction

Decisions under risk often involve payoffs that are not monetary (a medical doctor taking decisions about a patient, a lawyer in a legal trial, or the decision whether or not to pursue a healthy lifestyle, for example) but involve rewards of a different nature (professional success, reputation, health, etc.). In this paper we test whether the risk aversion measures elicited through lotteries, the most popular elicitation method according to [1] and one of the most popular for [2], are robust to different domains. Is risk aversion in lotteries constant across domains? If this is the case, the risk attitude can be considered a primitive parameter that characterizes an individual, regardless of the reward. On the contrary, if domain matters, then the subject’s behavior in monetary lotteries would not help to predict behavior when the rewards are of a different nature. On the other hand, when subjects face the same risky decisions but in different scenarios, the framing may affect the decisions even if the underlying lottery (probabilities and rewards) is the same. Our second objective will be to see if the way in which we present the information affects decision making under risk. If there are no differences, risk aversion parameters obtained in lottery scenarios could be extrapolated to other contexts. By contrast, if framing affects decision making risk attitudes measured in one scenario would not be valid to predict subjects’ behavior when the context is entirely different.

Elicitation techniques estimate risk attitudes through different mechanisms that involve a certain domain and framing (see [3] on the different elicitation methods). In this paper domain refers to the nature of the outcome (monetary payoffs, level of health, professional success…), and framing to the scenario through which risk attitudes are elicited (the content of the task presented to the subjects, e.g. a multiple price list lottery). Previous literature has found that risk attitudes are not robust to the elicitation method [1, 4–9] but the comparisons across elicitation techniques involve different rewards and different framings simultaneously so that it is not clear which is responsible for the lack of robustness. Our main objective is to separate the impact of domain and framing in risk attitude elicitation.

To disentangle domain and framing effects, subjects will face a series of multiple price list lotteries with a safe option (similar to [10]). Concerning the nature of the reward, we consider two different domains. In the first domain rewards for the lottery are monetary (*Money domain*). In the second domain the same lottery is rewarded with grade points (*Grade domain*). We chose grades as an incentive because they have been compared to monetary rewards by previous studies and both have been found to be relevant to enhance performance [11]. In order to identify framing effects, we again compare two different scenarios while keeping the domain constant (grade points). In the first framing, we elicit risk attitudes through a multiple price list with a safe option (*lottery framing*). In the second, while keeping the same implicit lottery structure, the options are presented as the decision whether to answer multiple-choice exam questions or omit (*exam framing*); to maintain the same lottery structure, the questions were chosen so that subjects did not know the answer and were therefore playing the same implicit lottery as in the *lottery framing*. We will therefore have a total of three treatments: grade, lottery and exam. Previous studies have compared paid lottery choices and hypothetical questions to risk behavior in different contexts [12, 13] or to contributions to a public good [14]. Our main contribution is an experimental design that allows to disentangle the effect of domain and framing simultaneously in a within subject design where all participants face all possible scenarios.

Our results show no differences in elicited risk aversion between the two domains (money domain or grade domain). However, framing matters: risk aversion is lower in the exam framing. Previous literature in multiple-choice tests [15, 16] found that males omit less questions, presumably because they are less risk averse compared to their female counterparts. However, we do not find any gender difference.

The paper is organized as follows. Section 2 contains the literature review, Section 3 describes the methods and research hypotheses, while Section 4 provides the details of the experimental design. In Section 5 we test, first, if changing the nature of the reward from money to grades (domain) affect their elicited risk preferences and, second, if framing affects the results; we also examine differential treatment effects by gender. Section 6 concludes.

## 2. Literature review

There are many different methods of risk preferences elicitation. [3] classify these methods in propensity, frequency and behavioral methods. In the first two, questionnaires about subjects’ daily activities or their views about risky prospects are used to estimate subjects’ risk attitudes; most of these questionnaires are hypothetical so that the subject’s answers do not affect rewards. On the contrary, behavioral methods for risk elicitation are not hypothetical, subjects have to select among different risky options with monetary implications. Previous literature has compared risk aversion estimations in and across the different elicitation methods. Some of these studies perform between-subject comparisons where each subject only faces one elicitation method [1, 5]. Others perform within-subject comparisons where each subject faces different elicitation methods ([3, 4, 6–9]. All of them find that the results are not robust to the elicitation method. These comparisons are performed across different techniques involving various reward schemes and framings simultaneously.

One important aspect to understand differences in elicited preferences could be the nature of the reward presented to subjects (domain). [12, 13, 17, 18] elicit risk preferences when the rewards vary (e.g. monetary vs health) and at least one of the rewards is hypothetical; all find that risk attitudes are not always constant across different domains. Notice that [19], find that hypothetical and monetary lotteries give different result when the framing is the same. By contrast, in [14] two different domains are considered: monetary and environmental, and subjects obtain money or trees planted on their behalf, respectively. In this comparison, one reward affects own profit (money) while the other takes the form of a contribution to the public good (environment). They find that subjects are more risk averse in the environmental domain. Finally, [11] compared how money versus extra class points influenced subjects in a task where participants pay to avoid risks. They found that the average amount participants would “pay” to avoid risk did not differ between the two forms of incentives. None of these papers compare risk attitudes when different personal rewards are at stake.

Another differential element between elicitation techniques is framing. [20–22] introduce changes to the usual framing in multiple price list (MPL) lotteries by varying different elements such as the order used to present the information or how the lotteries are explained to subjects. All find differences in elicited risk attitudes when information is presented in different formats. In addition, [23] also manipulate the framing of some lotteries, where the potential earnings make reference to a health context and subjects obtain monetary rewards by ensuring that their patients live in better conditions; different treatments frame the potential health of hypothetical patients differently (e.g. years in perfect health vs. hours of pain alleviated). They also find differences in elicited risk aversion across framings.

To sum up, previous literature has studied whether risk preferences are stable to different framings [20, 21], to different types of reward [14] or to simultaneous variations in domain and framing [3]. However, none of the studies perform a within subject analysis where the effect of domain and framing in risk preferences can be disentangled.

Elicited risk preferences may also be affected by subjects’ characteristics such as age [24]), having a family [25, 26] or gender. [27] also found that subjects are less risk averse when the money at stake is not theirs. Traditionally, females were believed to be more risk averse [2, 28, 29] but recent contributions find that this is not always the case [30–32]. [1] show that gender differences in risk attitudes depend on the elicitation method adopted. Differences are frequently found in some tasks, such as the ordered lottery selection task introduced by [29], whereas no gender differences are apparent in other contexts such as the multiple price list lotteries [33]. More generally, gender differences observed in some studies might be the result of particular features of the experimental design. For example, analyzing gender differences in prosociality, [34] find that differential treatment effects across genders may be due to the differential effect of framing. In this paper, we look at the possible differential effect that domain and framing may have on subjects with different gender, which may explain the fact that some elicitation methods produce gender differences while others do not.

## 3. Methods

Our first goal is to check if changing the domain (nature of the reward) of payoffs in a price list task might impact the elicited risk preferences. Notice that in this paper domain always refers to the argument of the utility function. To test this, we consider two different domains. In a lottery with a safe option (see [10]) rewards are monetary in the Money treatment (*Mo*), or grade points in a regular first year university course in the Points treatment (*Po*).

Our second aim is to test if framing affects risk aversion attitudes. Framing refers to the setting or situation faced by the subject in the experiment. Notice that we keep the argument of the utility function (domain) constant in both framing treatments (grade points). In order to identify framing effects, we create two different scenarios while keeping the domain constant (grade points). In the first framing, we elicit risk attitudes through an MPL lottery with a safe option. In the second, while keeping the same underlying lottery structure, the options are presented as the decision whether to answer exam questions or omit (*Ex* or Exam treatment). Table 1 describes the differences in domain and framing between treatments.

**Table 1:** Treatments.

	Domain	
Framing	Money	Points
Lottery	*Mo*	*Po*
Exam	-------	*Ex*

For each domain/framing we estimate the risk aversion coefficient assuming a CRRA utility function (constant relative risk aversion):

$$U\left(x\right)=\left\{\begin{array}{ll} \frac{x^{\left(1-r\right)}}{\left(1-r\right)}, & r>0,r\neq 1\\ \text{ln}\left(x\right), & r=1 \end{array}\right.$$

where *x* is the reward (either money or points in the exam) and the coefficient *r* is the parameter to be estimated; *r* measures the degree of risk aversion that is implicit in this function. The estimation is performed using maximum likelihood structural estimation [35, 36]. We use a Fetchner error specification corrected as in [37], to avoid the non-monotonicity of choice probabilities when *r* varies [37–39].

MPL lotteries have been criticized [5] for generating inconsistent observations (subjects may switch options more than once) that have to be excluded from the analysis. With a maximum likelihood structural estimation, we are able to overcome this issue and include all the decisions, since choices are rationalized by adding a stochastic component to model errors. Moreover, [40] point out that for subjects with high numeracy skills, such as the ones in this sample (all university students), MPL is an appropriate method due to the predictive accuracy of the task. [41] argue that preferences inferred through choice lists provide a better indicator of informed preferences compared to elicitation based on a single choice.

The maximum likelihood regression results will provide the treatment effects and this will allow us to test our hypotheses:

- *H1*: *Ceteris paribus*, *elicited risk aversion is different in the grades domain (treatment Po) than in the money domain (treatment Mo)*.

This hypothesis is based on previous findings that a change in hypothetical domains affects elicited risk attitudes ([12, 13, 17]. [14] also found that risk attitudes were not stable in two scenarios with real rewards, one where subjects earned money and another where they contributed to the environment with trees planted.

Treatments *Mo* and *Po* only differ in the nature of payoffs (domain), Money in *Mo* and grade points in *Po*; everything else is identical, including the terms of the lotteries.

- *H2*: *Ceteris paribus*, *elicited risk aversion is different in the lottery-framing (treatment Po) than in the exam-framing (treatment Ex)*.

Hypothesis 2 is formulated taking into account that previous literature analyzing small framing variations in MPL lotteries (e.g. [20, 21]) found that framing affects risk-taking behavior.

Note that in *Po* and *Ex* we keep the nature of the reward constant while we vary the framing, a lottery in *Po* and an exam in *Ex*.

- *H3*: *Domain or framing do not affect gender differences in risk aversion*.

This third hypothesis implies that any possible gender differences in risk aversion will remain constant across treatments; for example, if women are more risk averse than men in *Mo*, our hypothesis is that they will also be more risk averse in the other two treatments, *Po* and *Ex*. This hypothesis is supported by previous literature on framing such as [20, 23] who did not observe significant gender differences across treatments with different framings.

## 4. Experimental design

The experiment was performed with 249 first year students enrolled in the course *Introduction to Business* at the University of the Balearic Islands (Spain). The total number of students enrolled was 273. Participation was voluntary and 91% of the students decided to participate and thus gave oral consent to participate in the study. Students were invited to participate in the experiment after their first midterm exam. The only information they had regarding the experiment was the expected duration and that by participating they could increase their mark in the exam and earn some money. As specified in the syllabus and teaching guide, students can increase their marks along the course by participating in educational activities. In this particular activity, students familiarize themselves with the concepts of lottery and risk, which are later the subject matter of a lecture in financial decision making where we formally introduce the concept of risk aversion. It was a pen-and-paper experiment that consisted on three different tasks and a questionnaire. They needed to complete everything to obtain the points/money.

The experiment has a within-subject design, and all the subjects participate in all the 3 treatments in different orders. The treatments are as follows:

### 4.1. Money treatment (*Mo*)

Subjects face a lottery that consists of a series of 11 decisions (see Table 2) where outcomes are monetary. Two randomly selected subjects are paid for one randomly selected decision. The payoffs are expressed in ECUs (experimental currency units). Subjects are informed about the exchange rate: 1 ECU is equivalent to 5 Euros. Subjects can earn between 0 and 50 Euros in this task.

**Table 2:** Lotteries in Money treatment, *Mo*.

Decision	Option A	Option B
1	2 ECU	1/5 probability of winning 10 ECU, 4/5 probability of winning 0 ECU
2	2 ECU	1/5 probability of winning 10 ECU, 4/5 probability of winning 0.2 ECU
3	2 ECU	1/5 probability of winning 10 ECU, 4/5 probability of winning 0.4 ECU
4	2 ECU	1/5 probability of winning 10 ECU, 4/5 probability of winning 0.6 ECU
5	2 ECU	1/5 probability of winning 10 ECU, 4/5 probability of winning 0.8 ECU
6	2 ECU	1/5 probability of winning 10 ECU, 4/5 probability of winning 1 ECU
7	2 ECU	1/5 probability of winning 10 ECU, 4/5 probability of winning 1.2 ECU
8	2 ECU	1/5 probability of winning 10 ECU, 4/5 probability of winning 1.4 ECU
9	2 ECU	1/5 probability of winning 10 ECU, 4/5 probability of winning 1.6 ECU
10	2 ECU	1/5 probability of winning 10 ECU, 4/5 probability of winning 1.8 ECU
11	2 ECU	1/5 probability of winning 10 ECU, 4/5 probability of winning 2 ECU

Note: 1 ECU is equivalent to 5 Euros.

We use a multiple price list lottery composed of multiple choices where the risky option is increasing in expected value. There is always a safe option, so that subjects decide between a lottery and earning a safe amount [10]. The reason for choosing a multiple price list with a safe option is to be able to maintain the same underlying lottery structure when the framing is changed to a multiple-choice test where the safe option is to omit the question. With these variations, we can use the same underlying lottery in our three scenarios which makes risk attitudes comparable across treatments. In return, including a safe option does not allow to measure risk proneness.

In Table 2, Option A is the safe option, where subjects earn 10 Euros for sure if that decision is implemented. Option B is a series of ordered lotteries with increasing expected value, where subjects could earn 50 euros with a probability 0.2 or a lower amount (from 0 in decision 1 to 10 Euros in decision 11) with probability 0.8. Subjects made a choice between A and each lottery in column B, switching at some point from the safe option (A) to the risky option (B) as the probability of the good outcome in the risky lottery increases. The switching point captures their degree of risk aversion. For example, if subject 1 chooses column A five times and subject 2 chooses column A ten times, we infer that subject 2 is more risk averse than subject 1, since the former selected a higher number of safe options. Never choosing the risky option or switching from B to A are regarded as inconsistent choices; these decisions can be rationalized by adding a stochastic component to model errors.

### 4.2. Grade points treatment (*Po*)

This treatment has the same lottery structure as the Money treatment (*Mo*) but the reward pertains to a different domain: instead of earning money, subjects earn grade points for the final score. The midterm exam is graded over 100 and accounts for 25% of the final score of the course. Depending on their choice subjects could obtain between 0 and 10 extra points in the midterm exam. Note that the amount of points and money that subjects could potentially earn was not large. The Ethics Committee of the University of the Balearic Islands allowed our intervention (number 89CER18) on the condition that (a) the points at stake were extra points added to the final mark (we could not decrease scores), (b) these extra points did not represent more than the 10% of the value of the midterm exam. Students were aware that one of the decisions in this task or the following task would be implemented and the points obtained added to the exam. Notice that the points offered to students represented a maximum of a 2.5% of the final grade, which is not high but can still help to pass the course. Compared to the money they could earn it may seem low but, since only randomly selected participants were paid, the stakes in the two treatments are comparable.

Table 3 shows the multiple price list lottery. Again, choosing Option A is the safe option. Selecting Option A means that if this decision is implemented, 2 extra points will be added to the exam for sure. Meanwhile, column B contains a series of lotteries of increasing expected value where subjects can earn 10 points in the exam with probability 0.2 or a lower increasing amount (from 0 points in decision 1 to 2 points in decision 11) with probability 0.8.

**Table 3:** Lotteries in grade points treatment, *Po*.

Decision	Option A	Option B
1	2 points	1/5 probability of winning 10 points, 4/5 probability of winning 0 points
2	2 points	1/5 probability of winning 10 points, 4/5 probability of winning 0.2 points
3	2 points	1/5 probability of winning 10 points, 4/5 probability of winning 0.4 points
4	2 points	1/5 probability of winning 10 points, 4/5 probability of winning 0.6 points
5	2 points	1/5 probability of winning 10 points, 4/5 probability of winning 0.8 points
6	2 points	1/5 probability of winning 10 points, 4/5 probability of winning 1 point
7	2 points	1/5 probability of winning 10 points, 4/5 probability of winning 1.2 points
8	2 points	1/5 probability of winning 10 points, 4/5 probability of winning 1.4 points
9	2 points	1/5 probability of winning 10 points, 4/5 probability of winning 1.6 points
10	2 points	1/5 probability of winning 10 points, 4/5 probability of winning 1.8 points
11	2 points	1/5 probability of winning 10 points, 4/5 probability of winning 2 points

### 4.3. Exam treatment (*Ex*)

In this treatment, the lottery structure is the same as in treatments *Mo* and *Po*, but with an exam framing. Students were informed that one of the decisions made in this task or the previous one would be implemented and the points obtained added to the midterm exam grade. One out of the 22 decisions made in *Po* and *Ex* treatments was selected to reward subjects.

We replicate the same lottery structure in a multiple-choice test framing with questions that our subjects are very unlikely to know the answer to, so that we can control the probability of success. If students chose one of the 5 potential answers randomly, on average 20% of the answers would be right. In our case, 18% of the subjects guessed the correct answer. This number is very close to the theoretical 20%. See S2 Appendix for more information on subjects’ answering pattern. A subject was given a randomly chosen question with 5 potential answers and only one is correct (see Table 4 for a sample question of this treatment and S2 Appendix to see other questions in this treatment). The first task is to choose one correct answer (the question is the same and the answer is unique for all the lotteries). Then, they have to choose between the safe option (omit), earning 2 points for sure by refusing to give an answer, or decide to answer. This is done by circling *Yes* or *No* to answer the question in that particular decision. Circling *Yes* means answering, which implies playing a lottery since they do not know the correct answer, nor do they have partial knowledge regarding the answer. Therefore, this is the same as choosing Option B in the grade points treatment *Po*, where they earn 10 points with probability 0.2 (choosing randomly the correct answer) or answering incorrectly and earning a lower increasing amount (from 0 points in Decision 1 to 2 points in Decision 11) with probability 0.8. Circling *No* is equivalent to choosing Option A (safe option) in the grade points treatment *Po*.

**Table 4:** Exam treatment, *Ex*. Question: When was prospect theory first introduced by Kahneman and Tversky? a) 1980 b) 1979 c) 1978 d) 1977 e) 1976.

Decision	Omit (do not answer)	Right answer	Wrong answer	Circle YES if you would prefer to answer and NO if you would prefer to omit)
1	2 points	10 points	0 points	YES / NO
2	2 points	10 points	0.2 points	YES / NO
3	2 points	10 points	0.4 points	YES / NO
4	2 points	10 points	0.6 points	YES / NO
5	2 points	10 points	0.8 points	YES / NO
6	2 points	10 points	1 point	YES / NO
7	2 points	10 points	1.2 points	YES / NO
8	2 points	10 points	1.4 points	YES / NO
9	2 points	10 points	1.6 points	YES / NO
10	2 points	10 points	1.8 points	YES / NO
11	2 points	10 points	2 points	YES/ NO
Which answer is the correct one? (a, b, c, d or e)?				

The three treatments are equivalent in the probabilities of the lotteries involved. From money (*Mo*) to grade points (*Po*) we only change the nature of the reward and from grade points (*Po*) to exam (*Ex*) we only change the framing (lottery vs exam). We randomize the order of treatments to account for potential order effects; there are three different orders (see Table 5).

**Table 5:** Order of treatments.

Order	1^st^ task	2^nd^ task	3^rd^ task	Number of subjects
**0**	*Mo*	*Po*	*Ex*	69
**1**	*Po*	*Mo*	*Ex*	91
**2**	*Ex*	*Mo*	*Po*	89
**N**				249

## 5. Results

### 5.1. Descriptive statistics and non-parametric tests

Table 6 summarizes the main descriptive statistics. Note that our percentage of inconsistent subjects (*Mo*: 16%, *Po*: 17% and Ex: 14%) is similar to those in the literature: [33] find 13% inconsistent answers for American students, [40] find 8.5% of inconsistent subjects and [10] 15.6% in an multiple price list lottery. First, we are interested in the comparison between the first two treatments (*Mo* and *Po*). This comparison allows testing Hypothesis 1 (a change in domain changes elicited risk preferences). In *Mo* subjects face a lottery where they can earn money and in *Po* they face the same lottery but their reward are extra points in an exam. The difference between the amount of safe choices selected in *Mo* and *Po* by consistent subjects is not statistically significant (Mann-Whitney: *z = -1*.*254*, *p = 0*.*21*).

**Table 6:** Descriptive statistics for each treatment.

	*Mo*	*Po*	*Ex*
**# safe choices: mean**	5.314	5.631	5.014
**male**	5.313	5.663	4.682
**female**	5.315	5.611	5.234
**standard deviation**	2.298	2.184	3.007
**male**	2.409	2.204	3.091
**female**	2.231	2.180	2.942
**Number of subjects**	210	206	213
**% males**	36.94%	36.94%	36.94%
**Inconsistent subjects**	15.66%	17.27%	14.46%
**male**	9.78%	13.04%	7.61%
**female**	19.11%	19.75%	18.47%
**Strongly inconsistent subjects**	12.45%	13.25%	11.24%
**male**	8.7%	10.87%	5.43%
**female**	14.65%	14.65%	14.65%
**Always safe option**	3.21%	4.02%	3.21%
**male**	1.09%	2.17%	2.17%
**female**	4.46%	5.1%	3.82%

*Note*: *Strongly inconsistent subjects* are those who switch multiple times from safe to unsafe. *Always safe option* refers to subjects who chose the safe option in all the 11 decisions. *Inconsistent subjects* include strongly inconsistent and those who always chose the safe option. Inconsistent subjects were excluded from the sample used to calculate the mean safe choices.

Hypothesis 2 evaluates if framing modifies elicited risk preferences. In *Po* subjects face a lottery framing and in *Ex* they face an exam framing. The incentive in both cases was the same. We compare the amount of safe choices selected in these treatments by consistent subjects and observe significant differences between *Po* and *Ex* (Mann-Whitney: *z = 2*.*250; p = 0*.*024*). Subjects choose more safe options in *Po*.

Finally, Hypothesis 3 analyses differential effects of domain or framing by gender. If we compare the amount of safe choices made by male and female subjects across treatments we do not find significant gender differences in *Mo* treatment (Mann-Whitney: *z = 0*.*120; p = 0*.*905*), nor in *Po* (*z = -0*.*071; p = 0*.*944*) or in *Ex* (*z = 1*.*472; p = 0*.*141*). However, when we compare the behavior of individuals of the same gender in different treatments, we find that males behave differently in *Po* and *Ex* (*z = 2*.*09; p = 0*.*037*). We do not find significant differences in the amount of safe choices made by men or women across the rest of the treatments.

In the following sections we further analyze treatment effects.

### 5.2. The effect of domain: Grade vs money

To compare treatments *Mo* and *Po* we take all the observations where any of these treatments was performed first and hence, they were not affected by *Ex* treatment, i.e. orders 0 and 1 (see Table 5).

Table 7 presents the results of a maximum likelihood estimation of the risk aversion coefficient *r*, corrected for stochastic errors [37]. Even though there was random assignment, we have 1,518 observations where *Mo* came before *Po* and 2,002 observations where *Po* came before *Mo*. Due to the fact that the number of observations turned out not to be balanced, we introduce a dummy variable (*order*) to control for potential order effects in the regression; it has value 1 if *Po* was performed before *Mo* and 0 otherwise. A dummy variable named *treatmentPo* is added and has value 1 if the treatment is *Po* and 0 if the treatment is *Mo*. We also introduce a dummy variable (*male* with value 1 if the subject is a male and 0 otherwise) to control for gender effects. Finally, we add the interaction between gender and treatment (male*treatmentPo).

**Table 7:** The effect of domain. Maximum likelihood estimation of the risk aversion coefficient, *r*.

*r*	*Po-Mo*	*Po-Mo*
	(1)	(2)
*treatmentPo*	0.035 (0.025)	0.032 (0.0303)
*order*	0.002 (0.045)	-0.012 (0.046)
*male*		0.012 (0.057)
male*treatmentPo		0.029 (0.053)
*constant*	0.222*** (0.032)	0.205*** (0.063)
*mu*	0.106*** (0.006)	0.106*** (0.006)
Pseudo log likelihood	-860.953	-849.291
Prob>chi^2^	0.361	0.060
Observations	3,520	3,520
Control variables	NO	YES

*Note*: Controls (see definitions in S1 Appendix): *best group*, *worst group*, *English group*, *big town*, # *sitting exam*, *Erasmus*, *economics/business school* and *inconsistent*. The baseline treatment is the money lottery. The dependent variable is *r*. Standard errors (in parenthesis) are clustered by subject.

*p < 0.10,

** p < 0.05,

*** p < 0.01.

We can see that there are no order effects and the treatment is not significant. In addition, the coefficient associated to gender and the interaction of gender and treatment are not significant, gender differences in attitudes towards risk are constant when the domain varies (results hold if we perform the same regressions restricted only to male or female participants). These results hold whether we use control variables (column 2) or not (column 1).

**Result 1:** There are no differences in elicited risk aversion in the two domains considered (monetary and grade points).

This result contradicts previous findings by [12–14, 17] who find differences in risk taking attitudes when domains vary. One reason for the different findings is most of these papers both domain and framing change simultaneously. [12] compare self-reported risk attitudes in different contexts (e.g. health or financial decisions), so that not only domain in our sense (argument of the utility function) changes but also the framing (e.g. health or investment context).

[13] compare risk attitudes elicited through a nationwide questionnaire that includes questions in different contexts, to the results of an incentivized lottery with experimental subjects in a lab. They find that the measures are correlated and that the differences between them can be explained by context. They conclude that the risk measure elicited through a lottery can be replicated if subjects respond to many questions providing self-reported information in different contexts.

[17] elicits risk attitudes in the financial and environmental contexts using a multiple-price list experiment. Both contexts are hypothetical as subjects earnings are independent from the decisions made in the MPL experiments. She finds differences that could be due to domain or to the financial/environmental context.

These papers use at least one hypothetical scenario in their comparisons, while [14] compare a domain with individual monetary rewards to an environmental domain where the reward is an in-kind contribution to a public good (bee-friendly plants). The framing includes information about the decline of the bee population and the link between bee-friendly plants and the positive externality they generate. Our study adds to the literature by considering two different personal rewards, money or grade points.

In this paper we separate the effect of domain (the nature of the rewards) and the effect of framing. The variation in domain presented in this study turns out to be irrelevant (see Table 7) but, as we will show in the next section, framing affects elicited risk preferences.

### 5.3. The effect of framing

To compare treatments *Ex* and *Po* we take the orders 1 and 2. In order 1 subjects perform *Po* first and *Ex* last, and in order 2 they start with *Ex* and finish with *Po*. Since we have 3,003 observations in order 1 and 2,937 observations in order 2, we can conclude that the sample is balanced. Nevertheless, we control for any potential order effects.

Table 8 presents the results of the maximum likelihood estimation. The independent variables are *treatmentEx* (a dummy with value 1 if the treatment is *Ex*); *order* with value 1 if treatment *Ex* goes before treatment *Po* and 0 otherwise; *male*, equal to 1 if the subject is a male; and male*treatmentEx is the interaction between the variables *male* and *treatmentEx*. Models (1) and (2) take into account all the subjects in orders 1 and 2, while Models (3) to (6) further restrict the sample to males or females.

**Table 8:** The effect of framing. Maximum likelihood estimation of the risk aversion coefficient, *r*.

ρ	(1)	(2)	(3)	(4)	(5)	(6)
	Ex-Po	Ex-Po	Ex-Po	Ex-Po	Ex-Po	Ex-Po
			males	males	females	females
*treatmentEx*	-0.143*** (0.038)	-0.117** (0.047)	-0.169** (0.061)	-0.211*** (0.066)	-0.124*** (0.046)	-0.109** (0.049)
*order*	0.058 (0.050)	0.097* (0.050)	0.064 (0.082)	0.162* (0.085)	0.056 (0.062)	-0.070 (0.060)
*male*		-0.040 (0.062)				
*male* * *treatmentEx*		-0.101 (0.082)				
*constant*	0.267*** (0.036)	0.235*** (0.707)	0.244*** (0.069)	0.232** (0.103)	0.282*** (0.041)	0.186*** (0.092)
*mu*	0.126*** (0.006)	0.1125*** (0.006)	0.118*** (0.009)	0.115*** (0.008)	0.132*** (0.009)	0.147* (0.096)
Pseudo log likelihood	-1088.026	-1065.058	-415.554	-396.683	-668.808	-651.61
Prob>Chi^2^	0.000	0.000	0.011	0.002	0.020	0.002
Observations	3,960	3,960	1540	1540	2420	2420
Control variables	NO	YES	NO	YES	NO	YES

*Note*: Controls (see definitions in S1 Appendix): *best group*, *worst group*, *English group*, *big town*, # *sitting exam*, *Erasmus*, *economics/business school* and *inconsistent*. The baseline treatment is the Grade Points lottery *Po*. The dependent variable is *r*. Standard errors (in parenthesis) are clustered by subject.

*p < 0.10,

** p < 0.05,

*** p < 0.01.

Model (1) in Table 8 shows that the treatment is significant. Subjects are significantly less risk averse in an exam framing (*Ex*) compared to a lottery framing (*Po*) with the same underlying structure. The variable *order* is not significant at 5% In Model (2) we see that the variable male and the interaction between treatment *Ex* and gender is not significant. Overall, subjects show less risk aversion in Ex than Po.

**Result 2:** There are differences in the elicited risk aversion parameter between the two framings considered (lottery and a multiple-choice test).

In Models (3) and (4) of Table 8 the estimation is restricted to males (without and with controls, respectively). The numbers of male and female subjects are unbalanced in the sample (63% females). Note that for males, treatment *Ex* is highly significant (at 1%) and positive. The coefficient is quite large (-0.211). Males in treatment *Ex* are less risk averse than in treatment *Po*. Restricting the sample to females, Models (5) and (6), the coefficient associated to treatment *Ex*, (-0.109) is also significant (at 1%). The average treatment effect seems to be larger for males than females in the separate regressions, but the difference is not significant in the overall sample.

**Result 3:** There are no significant differences between males and females, both show more risk aversion in a lottery framing than in a multiple-choice test framing.

[1] find that in multiple price list lotteries both genders display a similar behavior. They conduct a meta-study with 20 papers using MPL lotteries and find no gender differences in 15 of these studies, mixed results in 2 and gender differences in 3 of the studies. None of the studies finding gender differences used the basic [33] MPL treatment. Two of these three papers used a [33] MPL elicitations task. [42] used a low stake [33] task, while [40] run a high stake (x20) [33] MPL. The third study finding gender differences, [43] used a non-incentivised version with less choices (9).

According to the literature studying behavior in multiple choice tests with correction for guessing [15, 16, 44, 45], males show less risk aversion in exams and answer more questions than females. However, we did not find significant gender differences.

### 5.4. Subjects’ behavior in the exam framing

The impact of framing could act through the channel of probability perception. To explore this possibility, let’s analyze the answering patterns in *Ex* treatment. The test makers had placed the correct answer in positions A, B, C, D or E randomly (see S2 Appendix for the different questions and answers in the *Ex* treatment). Subjects had no knowledge, so the probability of each alternative A, B, C, D or E being the correct answer was the same. Under these conditions, if the subject decides to take the lottery of answering the question, we would expect a random choice. However, the pattern of the answers was not random.

Had subjects answered randomly, each answer (A, B, C, D and E) should have been selected 20% of the times. A χ^2^ test against the theoretical distribution (*p = 0*.*000*) indicates that subjects did not answer randomly the questions in the *Ex* treatment. This result holds for males (*p = 0*.*000*) and females (*p = 0*.*000*). We can rationalize this through two different mechanisms: focal points or miscalibration of probabilities.

**Fig 1:**
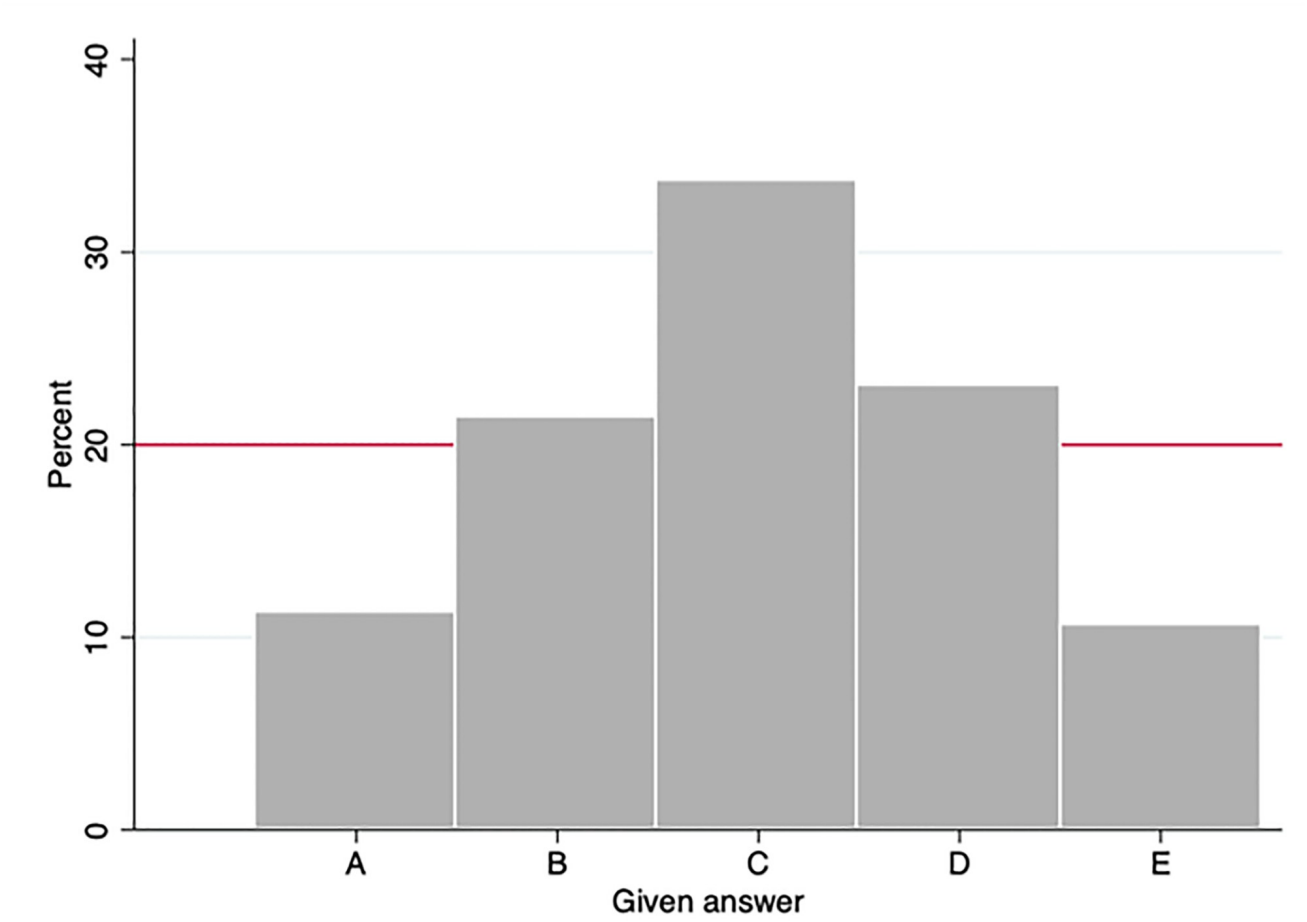
Answers provided by subjects in *Ex* treatment.

In Fig 1 and Table 9 we see that subjects exhibit a preference towards the middle option, answer C (33% of the cases). The first explanation is that when intending to choose randomly, the item in the middle, option C, is a focal point. In this case, subjects who are guessing the answer are attracted to this focal point and choose it more frequently than 1/5 of the times. If the focal point explanation is correct, the elicited risk aversion coefficient would be the same as in the MPL lotteries, that is, subjects perceive the probabilities of each option as being identical but are attracted by the focal point when guessing. Notice that the focal point theory is in line with the *central tendency bias*: when subjects have to estimate or choose among different quantities they tend to exhibit a bias towards the mean of the distribution [46–49].

**Table 9:** Frequency of each answer.

Given answers	Percentage	χ^2^ against theor. distribution
**A**	11.24%	p = 0.000
**B**	21.35%	p = 0.142
**C**	33.71%	p = 0.000
**D**	23.03%	p = 0.000
**E**	10.67%	p = 0.000

Previous literature in multiple-choice tests also allows to anticipate this as a plausible behavior in the Exam treatment. [50] find that subjects who do not know the answer in a multiple-choice test tend to randomly select the middle options (in their terminology, there is a *middle bias* or *edge aversion*; see also [51, 52]. Interestingly, they also find that test makers have a bias against the edges; when asked to write a single question with four alternatives, around 80% choose to place the correct answer in one of the two middle positions. If test takers believe that test makers have such a bias, then it is an optimal response for the test takers to show a middle bias. If this explanation (miscalibration explanation) is correct, the elicited risk aversion coefficient would be different from the one in the MPL lotteries, because subjects perceived the probabilities of each option as being distinct, higher for the middle options and lower for the edges. Note that in this case lotteries in the exam are viewed as less risky and therefore they are more likely to be taken. By contrast, if subjects are answering randomly (focal point explanation) we should expect the same elicited risk aversion coefficient as in MPL lotteries independent of their answering pattern. A similar argument is put forward in [53]. Entrepreneurs take higher risks not because they have different risk preferences than the rest of the population but because their perceived (or actual) probability of success is higher (see also [54]). From Table 8, we see in columns (1) and (2) that the effect of treatment *Ex* is negative and significant, which would favor the first explanation: subjects are attracted by the middle option because they think it has higher probability (miscalibration explanation).

Furthermore, framing may trigger several behavioral biases and lead to a miscalibration of the probability of choosing the correct answer. In our case, when comparing a lottery where the probabilities are explicit, to a different framing where the probabilities are the same but are not made explicit, we may find that behavior is different even though subjects’ risk preferences are stable. The exam framing may trigger the illusion of control [55], or an excessive optimism or overconfidence that may produce a distortion of the real probabilities of success. “The illusion of control [56] is concerned with greater confidence in one’s predictive ability or in a favorable outcome when one has a higher degree of personal involvement, even when one’s involvement is not actually relevant.” [55].

It is worth noting that in our context overconfidence is understood as overestimation of one’s actual knowledge [57]; however, in framing overconfidence is mitigated by the fact that the subjects had no knowledge on the questions, so it is unlikely that they could be overestimating their knowledge.

Therefore, the illusion of control or the overestimation of their abilities in the hide-and-seek game with the test maker (see [52]) leads to overestimation of their probability of guessing the right answer and thus to a different behavior even though the underlying lotteries are the same in treatments *Ex* and *Po*.

## 6. Conclusions

In this paper we analyze if elicited risk preferences are stable under changes in domain or framing. This issue is important because the elicitation methods used in the literature differ in the nature of the reward (domain) and/or the scenarios presented to subjects (framing). Ideally, we would like the results to be robust to domain or framing as that would render the results comparable across different elicitation methods. Our experimental evidence indicates that elicited preferences do depend on framework, while they seem to be independent of domain. This result allows reconciling some contradictory outcomes in the literature. Differences in elicited risk preferences probably come from the differential effect of framing, which may be confounded with different risk aversion.

We chose two different domains for the rewards, money and grades, that have been found relevant in previous literature. We do not find significant differences between subjects’ behavior in these two particular domains. Our findings are different from previous results because we separate domain, interpreted as the nature of the rewards, from the framing of the experiment. This differentiation has not been made in the literature and we find that it is an important one. Another difference is that we compare two personal incentives (money vs. grades) while previous literature has compared other scenarios such as monetary to hypothetical individual earnings or to contributions to a public good. Our result finding that domain is irrelevant, is in line with [11] who find that monetary and grade incentives generate no differences in a game where participants need to pay to avoid a risk. Further research is needed to show whether these results hold under different elicitation rewards and methods where for example risk proneness can be captured.

For differences in framing, we compare a modified multiple price list framing with a safe option to another where subjects face an identical underlying lottery but in an exam framing. We find that there are differences in elicited risk attitudes between these treatments. Framing may trigger behavioral biases (illusion of control, overoptimism) that affect the subjective probabilities of the lotteries. In our case subjects show more risk aversion in a lottery framing than in an exam framing.

Our results show that framing may trigger behavioral biases that can change the outcome of risk elicitation tasks. Future work should focus on the nature of the behavioral biases involved in framing and the implications for elicited risk attitudes in different contexts.

## Supporting information

S1 Data (ZIP)

S1 Appendix (DOCX)

S2 Appendix Questions in the exam treatment. (DOCX)

S3 Appendix Instructions. (DOCX)

## References

[1] Crosetto P, Filippin A. A theoretical and experimental appraisal of four risk elicitation methods. Experimental Economics. 2016 Sep;19(3):613–41.

[2] Charness G, Gneezy U. Strong evidence for gender differences in risk taking. Journal of Economic Behavior & Organization. 2012 Jun 1;83(1):50–8.

[3] Frey R, Pedroni A, Mata R, Rieskamp J, Hertwig R. Risk preference shares the psychometric structure of major psychological traits. Science advances. 2017 Oct 1;3(10):e1701381. doi: 10.1126/sciadv.1701381 28983511 PMC5627985

[4] Reynaud A, Couture S. Stability of risk preference measures: results from a field experiment on French farmers. Theory and decision. 2012 Aug 1;73(2):203–21.

[5] Charness G, Gneezy U, Imas A. Experimental methods: Eliciting risk preferences. Journal of Economic Behavior & Organization. 2013 Mar 1;87:43–51.

[6] Deck C, Lee J, Reyes JA, Rosen CC. A failed attempt to explain within subject variation in risk taking behavior using domain specific risk attitudes. Journal of Economic Behavior & Organization. 2013 Mar 1;87:1–24.

[7] Menkhoff L, Sakha S. Estimating risky behavior with multiple-item risk measures. Journal of Economic Psychology. 2017 Apr 1;59:59–86.

[8] Zhou W, Hey J. Context matters. Experimental economics. 2018 Dec;21(4):723–56. doi: 10.1007/s10683-017-9546-z 30459523 PMC6223722

[9] Holzmeister F, Stefan M. The risk elicitation puzzle revisited: Across-methods (in) consistency?. Experimental Economics. 2021 Jun;24(2):593–616. doi: 10.1007/s10683-020-09674-8 34776759 PMC8550567

[10] Crosetto P., & Filippin A. (2017). Safe options induce gender differences in risk attitudes.

[11] Kruse J. B., & Thompson M. A. (2001). A comparison of salient rewards in experiments: Money and class points. *Economics Letters*, 74(1), 113–117.

[12] Weber EU, Blais AR, Betz NE. A domain‐specific risk‐attitude scale: Measuring risk perceptions and risk behaviors. Journal of behavioral decision making. 2002 Oct;15(4):263–90.

[13] Dohmen T, Falk A, Huffman D, Sunde U, Schupp J, Wagner GG. Individual risk attitudes: Measurement, determinants, and behavioral consequences. Journal of the european economic association. 2011 Jun 1;9(3):522–50.

[14] Ioannou CA, Sadeh J. Time preferences and risk aversion: Tests on domain differences. Journal of Risk and Uncertainty. 2016 Aug 1;53(1):29–54.

[15] Ramos I, Lambating J. Risk taking: Gender differences and educational opportunity. School Science and Mathematics. 1996 Feb;96(2):94–8.

[16] Iriberri N, Rey-Biel P. Competitive pressure widens the gender gap in performance: Evidence from a two-stage competition in mathematics. The Economic Journal. 2019 May 1;129(620):1863–93.

[17] Riddel M. Comparing risk preferences over financial and environmental lotteries. Journal of Risk and Uncertainty. 2012 Oct;45(2):135–57.

[18] Festjens A., Bruyneel S., Diecidue E., & Dewitte S. (2015). Time-based versus money-based decision making under risk: An experimental investigation. *Journal of Economic Psychology*, 50, 52–72.

[19] García-Gallego A, Georgantzís N, Jaramillo Gutiérrez A, Parravano M. The lottery-panel task for bi-dimensional parameter-free elicitation of risk attitudes. CISC.

[20] Lévy-Garboua L, Maafi H, Masclet D, Terracol A. Risk aversion and framing effects. Experimental Economics. 2012 Mar;15(1):128–44.

[21] Loomes G, Pogrebna G. Measuring individual risk attitudes when preferences are imprecise. The Economic Journal. 2014 May 1;124(576):569–93.

[22] Estepa-Mohedano L, Espinosa MP. Comparing risk elicitation in lotteries with visual or contextual framing aids. MPRA paper 108440. 2021.

[23] Arrieta A, García‐Prado A, González P, Pinto‐Prades JL. Risk attitudes in medical decisions for others: an experimental approach. Health Economics. 2017 Dec;26:97–113. doi: 10.1002/hec.3628 29285873

[24] Banks J, Bassoli E, Mammi I. Changing attitudes to risk at older ages: The role of health and other life events. Journal of Economic Psychology. 2020 Aug 1;79:102208.

[25] Lejarraga T, Frey R, Schnitzlein DD, Hertwig R. No effect of birth order on adult risk taking. Proceedings of the National Academy of Sciences. 2019 Mar 26;116(13):6019–24. doi: 10.1073/pnas.1814153116 30858316 PMC6442587

[26] Görlitz K, Tamm M. Parenthood, risk attitudes and risky behavior. Journal of Economic Psychology. 2020 Aug 1;79:102189.

[27] Chakravarty S, Harrison GW, Haruvy EE, Rutström EE. Are you risk averse over other people’s money?. Southern Economic Journal. 2011 Apr;77(4):901–13.

[28] Croson R, Gneezy U. Gender differences in preferences. Journal of Economic literature. 2009 Jun;47(2):448–74.

[29] Eckel CC, Grossman PJ. Differences in the economic decisions of men and women: Experimental evidence. Handbook of experimental economics results. 2008 Jan 1;1:509–19.

[30] Booth AL, Nolen P. Gender differences in risk behaviour: does nurture matter?. The economic journal. 2012 Feb 1;122(558):F56–78.

[31] Booth A, Cardona-Sosa L, Nolen P. Gender differences in risk aversion: do single-sex environments affect their development?. Journal of economic behavior & organization. 2014 Mar 1;99:126–54.

[32] Lemaster P, Strough J. Beyond Mars and Venus: Understanding gender differences in financial risk tolerance. Journal of Economic Psychology. 2014 Jun 1;42:148–60.

[33] Holt CA, Laury SK. Risk aversion and incentive effects. American economic review. 2002 Dec;92(5):1644–55.

[34] Espinosa MP, Kovářík J. Prosocial behavior and gender. Frontiers in behavioral neuroscience. 2015 Apr 14;9:88. doi: 10.3389/fnbeh.2015.00088 25926783 PMC4396499

[35] Harrison GW. Maximum likelihood estimation of utility functions using Stata. University of Central Florida, Working Paper. 2008 May:06–12.

[36] Harrison GW, Rutström EE. Risk aversion in the laboratory. InRisk aversion in experiments 2008 Jun 3. Emerald Group Publishing Limited.

[37] Harrison G. W., Lau M. I., & Yoo H. I. (2020). Risk attitudes, sample selection, and attrition in a longitudinal field experiment. *Review of Economics and Statistics*, 102(3), 552–568.

[38] Wilcox N. T. (2011). ‘Stochastically more risk averse:’A contextual theory of stochastic discrete choice under risk. *Journal of Econometrics*, 162(1), 89–104.

[39] Apesteguia J., & Ballester M. A. (2018). Monotone stochastic choice models: The case of risk and time preferences. *Journal of Political Economy*, 126(1), 74–106.

[40] Dave C, Eckel CC, Johnson CA, Rojas C. Eliciting risk preferences: When is simple better?. Journal of Risk and Uncertainty. 2010 Dec;41(3):219–43.

[41] Freeman DJ, Mayraz G. Why choice lists increase risk taking. Experimental Economics. 2019 Mar;22(1):131–54.

[42] Agnew JR, Anderson LR, Gerlach JR, Szykman LR. Who chooses annuities? An experimental investigation of the role of gender, framing, and defaults. American Economic Review. 2008 May;98(2):418–22.

[43] Branas-Garza P, Rustichini A. Organizing effects of testosterone and economic behavior: not just risk taking. PloS one. 2011 Dec 29;6(12):e29842. doi: 10.1371/journal.pone.0029842 22242144 PMC3248440

[44] Baldiga K. Gender differences in willingness to guess. Management Science. 2014 Feb;60(2):434–48.

[45] Espinosa MP, Gardeazabal J. The Gender-bias Effect of Test Scoring and Framing: A Concern for Personnel Selection and College Admission. The BE Journal of Economic Analysis & Policy. 2020 Jul 1;20(3).

[46] Poulton EC. Models for biases in judging sensory magnitude. Psychological bulletin. 1979 Jul;86(4):777. 482484

[47] Huttenlocher J, Hedges LV, Vevea JL. Why do categories affect stimulus judgment?. Journal of experimental psychology: General. 2000 Jun;129(2):220. doi: 10.1037//0096-3445.129.2.220 10868335

[48] Crosetto P, Filippin A, Katuščák P, Smith J. Central tendency bias in belief elicitation. Journal of Economic Psychology. 2020 Jun 1;78:102273.

[49] Jazayeri M, Shadlen MN. Temporal context calibrates interval timing. Nature neuroscience. 2010 Aug;13(8):1020–6. doi: 10.1038/nn.2590 20581842 PMC2916084

[50] Attali Y, Bar‐Hillel M. Guess where: The position of correct answers in multiple‐choice test items as a psychometric variable. Journal of Educational Measurement. 2003 Jun;40(2):109–28.

[51] Bar-Hillel M, Attali Y. Seek whence: Answer sequences and their consequences in key-balanced multiple-choice tests. The American Statistician. 2002 Nov 1;56(4):299–303.

[52] Bar-Hillel M. Position effects in choice from simultaneous displays: A conundrum solved. Perspectives on Psychological Science. 2015 Jul;10(4):419–33. doi: 10.1177/1745691615588092 26177945

[53] Weber EU, Hsee C. Cross-cultural differences in risk perception, but cross-cultural similarities in attitudes towards perceived risk. Management science. 1998 Sep;44(9):1205–17.

[54] Cooper AC, Woo CY, Dunkelberg WC. Entrepreneurs’ perceived chances for success. Journal of business venturing. 1988 Mar 1;3(2):97–108.

[55] Charness G, Gneezy U. Portfolio choice and risk attitudes: An experiment. Economic Inquiry. 2010 Jan;48(1):133–46.

[56] Langer EJ. The illusion of control. Journal of personality and social psychology. 1975 Aug;32(2):311.

[57] Moore DA, Healy PJ. The trouble with overconfidence. Psychological review. 2008 Apr;115(2):502. doi: 10.1037/0033-295X.115.2.502 18426301

