# Supplementary material for: The effect of domain and framing on elicited risk aversion
Source: PLoS One. 2022 Sep 26;17(9):e0267696. doi: 10.1371/journal.pone.0267696 (PMC9512169; doi:10.1371/journal.pone.0267696)
Supplement: S1 Appendix — (DOCX) [file pone.0267696.s002.docx]

**Appendix A**

**Table A1**: Definition of control variables used in Tables 7, 8 and 9.

| **Variable** | **Explanation** |
| --- | --- |
| *male* | 1 if male, 0 if female |
|  |  |
| *worst group* | 1 if the student belongs to the group with lower marks; 0 otherwise.  Students in this group got significantly lower grades in their A-levels  (mean 6.13). The university assigns students to groups taking into  account their preferred timetable, which leads to unbalanced groups  in terms of grades. |
| *best group* | 1 if the student belongs to the group with higher marks; 0 otherwise.  These students got significantly higher grades in their A-levels  (mean 7.40). The university assigns students to groups taking into  account their preferred timetable, which leads to unbalanced groups  in terms of grades. E |
| *English group* | 1 if the subject did the task in English; 0 if the task was in Spanish. |
|  |  |
| *big town* | 1 if the subject attended high school in a town of more than  100,000 inhabitants; 0 otherwise |
|  |  |
| *#sitting exam* | Number of times each subject faced that exam (from 1 to 9), 1 if  it is the 1^st^ sitting of the exam; 2 if is the second; etc. |
| *Erasmus* | 1 if the student was enrolled in an Erasmus program; 0 otherwise. |
| *economics/business*  *inconsistent* | 1 if the subject is a student of Economics or Business; 0 if the student  comes from Tourism Studies.  1 if the subject was inconsistent in any task; 0 otherwise |

**Table A2**: Summary statistics for the control variables used in Tables 7, 8 and 9.

|  | **Mean** | **St. Dev.** | **Min.** | **Max.** | **Obs.** |
| --- | --- | --- | --- | --- | --- |
| **Variable** |  |  |  |  |  |
| *male* | 0.369 | 0.483 | 0 | 1 | 249 |
| *worst group* | 0.133 | 0.339 | 0 | 1 | 249 |
| *best group* | 0.201 | 0.4 | 0 | 1 | 249 |
| *English group* | 0.606 | 0.489 | 0 | 1 | 249 |
| *Big town* | 0.574 | 0.494 | 0 | 1 | 249 |
| *#sitting exam* | 1.477 | 1.13 | 0 | 9 | 249 |
| *Erasmus* | 0.1 | 0.301 | 0 | 1 | 249 |
| *economics/business* | 0.096 | 0.295 | 0 | 1 | 249 |
| *inconsistent* | 0.158 | 0.365 | 0 | 1 | 249 |
