# Supplementary material for: The effect of domain and framing on elicited risk aversion
Source: PLoS One. 2022 Sep 26;17(9):e0267696. doi: 10.1371/journal.pone.0267696 (PMC9512169; doi:10.1371/journal.pone.0267696)
Supplement: S2 Appendix — (DOCX) [file pone.0267696.s003.docx]

**Appendix B: Questions in the Exam treatment**

**Set of questions:**

1. When was prospect theory first introduced by Kahneman and Tversky? (correct answer: b)

a) 1980

b) 1979

c) 1978

d) 1977

e) 1976

1. Who won the Economics Nobel prize in 1980? (correct answer: d)

a) James Meade

b) James Tobin

c) Arthur Lewis

d) Lawrence Klein

e) George Stigler

1. When did Abraham Maslow publish his famous paper “A theory of human motivation”? (correct answer: a)

a) 1943

b) 1944

c) 1945

d) 1946

e) 1947

1. The impossibility theorem was introduced by… (correct answer: e)

a) Friedrich Hayek

b) James Meade

c) Theodore Schultz

d) Simon Kuznets

e) Kenneth Arrow

1. Which of the following was a relevant economist… (correct answer: e)

a) Michael Brown

b) Stanley Cohen

c) James Frank

d) Johannes Stark

e) Richard Stone

**Table B1**: Descriptive statistics of the answers in Task N (multiple choice exam)

|  | **Q1** | **Q2** | **Q3** | **Q4** | **Q5** |
| --- | --- | --- | --- | --- | --- |
| **Number of subjects** | 79 | 41 | 32 | 46 | 51 |
| **Right answer** | B | D | A | E | E |
| **% Right answers** | 26.92% | 21.95% | 12.9% | 11.85% | 7.84% |
| **males** | 29.62%  [27] | 23.53%  [17] | 23.07%  [13] | 15.38%  [13] | 9.52%  [21] |
| **females** | 25.49%  [52] | 20.83%  [24] | 5.26%  [19] | 12.12%  [33] | 6.67%  [30] |
| **% C’s (focal point)** | 21.79% | 51.21% | 31.25% | 45.65% | 15.68% |
| **males** | 33% | 35.29% | 30.76% | 23.08% | 19.04% |
| **females**  **% of B’s, C’s, D’s**  **(middle bias)**  **males**  **females** | 15.68%  80.77%  96.27%  72.55% | 62.5%  87.8%  76.47%  95.83% | 31.58%  68.75%  53.85%  78.95% | 54.55%  73.91%  61.54%  78.79% | 6.67%  72.55%  66.67%  76.67% |

*Note*: number of male and female subjects in brackets.
