# Supplementary material for: The effect of domain and framing on elicited risk aversion
Source: PLoS One. 2022 Sep 26;17(9):e0267696. doi: 10.1371/journal.pone.0267696 (PMC9512169; doi:10.1371/journal.pone.0267696)
Supplement: S3 Appendix — (DOCX) [file pone.0267696.s004.docx]

**Appendix C: Instructions**

**You are taking part in an extra post-exam task. Please read the following instructions carefully. Depending upon the decisions you make you can earn up to an extra point in the exam (in Tasks P and N) and some amount of money (in Task M). It is extremely important that you read the instructions carefully.**

**Absolutely no communication is allowed. Please address any questions you might have to us directly. Any violation of this rule will lead to the exclusion of the person from all the tasks.**

**Thank you for participating and good luck!!**

**Instructions Task M:**

You will make eleven choices in the table below: each time you will have to **choose between Option A and Option B**.

Once all the tasks have been submitted, we will **randomly select two students**, **who will be paid for one randomly selected decision**. If you are selected, we will randomly determine your money earnings for the option you chose for that decision. Earnings will be paid before Easter. Take into account that **1 ECU (economic currency unit) is equal to 5 Euros**, that is, the earnings obtained are multiplied by 5; for example, if you obtained 10 ECU you earn 50 Euros.

Please look at the empty boxes on the right hand side of the table below. You will have to write a decision, A or B in each of these boxes. Do not forget to fill out each box, otherwise the task will not be valid, and you will not get any earnings. For example, in the first choice you have to choose between earning 2 ECU for sure (Option A) or earning 10 ECU with probability 1/5 or obtaining 0 ECU with probability 4/5 (Option B).

**Which option do you prefer?**

| **Option A** | **Option B** | **Selected option**  **(A or B)** |
| --- | --- | --- |
| 2 ECU | 1/5 of winning 10 ECU, 4/5 of winning 0 ECU |  |
| 2 ECU | 1/5 of winning 10 ECU, 4/5 of winning 0,2 ECU |  |
| 2 ECU | 1/5 of winning 10 ECU, 4/5 of winning 0,4 ECU |  |
| 2 ECU | 1/5 of winning 10 ECU, 4/5 of winning 0,6 ECU |  |
| 2 ECU | 1/5 of winning 10 ECU, 4/5 of winning 0,8 ECU |  |
| 2 ECU | 1/5 of winning 10 ECU, 4/5 of winning 1 ECU |  |
| 2 ECU | 1/5 of winning 10 ECU, 4/5 of winning 1,2 ECU |  |
| 2 ECU | 1/5 of winning 10 ECU, 4/5 of winning 1,4 ECU |  |
| 2 ECU | 1/5 of winning 10 ECU, 4/5 of winning 1,6 ECU |  |
| 2 ECU | 1/5 of winning 10 ECU, 4/5 of winning 1,8 ECU |  |
| 2 ECU | 1/5 of winning 10 ECU, 4/5 of winning 2 ECU |  |

**Instructions Task P:**

You will make eleven choices in the table below: each time you will have to **choose between Option A and Option B.** Once all the tasks have been submitted, **we will randomly select one decision** from Tasks P and N, **and you will obtain the extra points depending on the choice you made in that decision.**

Please look at the empty boxes on the right hand side of the table below. You will have to write a decision, A or B in each of these boxes. Do not forget to fill out each box, otherwise the task will not be valid and you will not get any extra points. For example, in the first decision you have to choose between obtaining for sure 2 extra points to be added to the exam’s grade (option A) or obtaining 10 extra points with probability 1/5 or 0 extra points with probability 4/5 (option B).

**Which option do you prefer?**

| **Option A** | **Option B** | **Selected option**  **(A or B)** |
| --- | --- | --- |
| 2 points | 1/5 of winning 10 points, 4/5 of winning 0 points |  |
| 2 points | 1/5 of winning 10 points, 4/5 of winning 0,2 points |  |
| 2 points | 1/5 of winning 10 points, 4/5 of winning 0,4 points |  |
| 2 points | 1/5 of winning 10 points, 4/5 of winning 0,6 points |  |
| 2 points | 1/5 of winning 10 points , 4/5 of winning 0,8 points |  |
| 2 points | 1/5 of winning 10 points, 4/5 of winning 1 point |  |
| 2 points | 1/5 of winning 10 points, 4/5 of winning 1,2 points |  |
| 2 points | 1/5 of winning 10 points, 4/5 of winning 1,4 points |  |
| 2 points | 1/5 of winning 10 points, 4/5 of winning 1,6 points |  |
| 2 points | 1/5 of winning 10 points, 4/5 of winning 1,8 points |  |
| 2 points | 1/5 of winning 10 points, 4/5 of winning 2 points |  |

**Instructions Task N:**

In this task you will have **to answer a question for which we offer 5 alternative answers (only one is correct)**. In the table there are **11 different ways to evaluate the question**; for example, giving 10 points for a right answer, 2 points for not answering and 0,6 points for a wrong answer. For each of these 11 different ways to grade the question you have to **decide if you prefer to answer the question or not (selecting YES or NO in the table)**. **At the end of the table you will have to state your preferred answer** (a, b, c, d or e). You do have to answer the question for this task to be valid.

Once all the tasks have been submitted, **we will randomly select one decision** from Tasks P and N **and you will obtain extra points depending on the choice you made in that decision.**

Please look at the last column on the right hand side of the table below. If you circle **YES** that means that you would **prefer to answer** the question, if you circle **NO,** that means that you would **prefer to leave it blank (omit)**. Do not forget to fill out each box, otherwise the task will not be valid and you will not get any extra points. For example, if the last option to grade the question is randomly selected and you chose to omit (wrote NO in the box) you will obtain 2 points; if you chose to answer (wrote YES in the box) and answered correctly, you will obtain 10 extra points and if you were wrong you will obtain 2 extra points.

**Question: When was prospect theory first introduced by Kanheman and Tversky?**

a) 1980

b) 1979

c) 1978

d) 1977

e) 1976

**Options to grade this question.** **Would you answer?**

| Omit (do not answer) | Right answer | Wrong answer | Circle YES if you would prefer to answer and NO if you would prefer to omit) |
| --- | --- | --- | --- |
| 2 points | 10 points | 0 points | YES / NO |
| 2 points | 10 points | 0.2 points | YES / NO |
| 2 points | 10 points | 0.4 points | YES / NO |
| 2 points | 10 points | 0,6 points | YES / NO |
| 2 points | 10 points | 0.8 points | YES / NO |
| 2 points | 10 points | 1 point | YES / NO |
| 2 points | 10 points | 1.2 points | YES / NO |
| 2 points | 10 points | 1.4 points | YES / NO |
| 2 points | 10 points | 1.6 points | YES / NO |
| 2 points | 10 points | 1.8 points | YES / NO |
| 2 points | 10 points | 2 points | YES/ NO |
| Which answer is | the correct one? | (a, b, c, d or e)? |  |
